# Bacteriophages Are Good Estimators of Human Viruses Present in Water

**DOI:** 10.3389/fmicb.2021.619495

**Published:** 2021-05-03

**Authors:** Elisenda Ballesté, Anicet R. Blanch, Javier Mendez, Laura Sala-Comorera, Leena Maunula, Silvia Monteiro, Andreas H. Farnleitner, Andreas Tiehm, Joan Jofre, Cristina García-Aljaro

**Affiliations:** ^1^Departament de Genètica, Microbiologia i Estadística, Universitat de Barcelona, Barcelona, Spain; ^2^Department of Food Hygiene and Environmental Health, Faculty of Veterinary Medicine, University of Helsinki, Helsinki, Finland; ^3^Laboratório Analises, Instituto Superior Tecnico, Universidade Lisboa, Lisbon, Portugal; ^4^Institute of Chemical, Environmental and Bioscience Engineering, Research Group Environmental Microbiology and Molecular Diagnostics 166/5/3, TU Wien, Vienna, Austria; ^5^Research Division Water Quality and Health, Karl Landsteiner University of Health Sciences, Krems an der Donau, Austria; ^6^Department of Microbiology and Molecular Biology, DVGW-Technologiezentrum Wasser, Karlsruhe, Germany

**Keywords:** *Bacteroides*, bacteriophages, indicators, fecal pollution, crAssphage, enteric viruses, norovirus, adenovirus

## Abstract

The detection of fecal viral pathogens in water is hampered by their great variety and complex analysis. As traditional bacterial indicators are poor viral indicators, there is a need for alternative methods, such as the use of somatic coliphages, which have been included in water safety regulations in recent years. Some researchers have also recommended the use of reference viral pathogens such as noroviruses or other enteric viruses to improve the prediction of fecal viral pollution of human origin. In this work, phages previously tested in microbial source tracking studies were compared with norovirus and adenovirus for their suitability as indicators of human fecal viruses. The phages, namely those infecting human-associated *Bacteroides thetaiotaomicron* strain GA17 (GA17PH) and porcine-associated *Bacteroides* strain PG76 (PGPH), and the human-associated crAssphage marker (crAssPH), were evaluated in sewage samples and fecal mixtures obtained from different animals in five European countries, along with norovirus GI + GII (NoV) and human adenovirus (HAdV). GA17PH had an overall sensitivity of ≥83% and the highest specificity (>88%) for human pollution source detection. crAssPH showed the highest sensitivity (100%) and specificity (100%) in northern European countries but a much lower specificity in Spain and Portugal (10 and 30%, respectively), being detected in animal wastewater samples with a high concentration of fecal indicators. The correlations between GA17PH, crAssPH, or the sum of both (BACPH) and HAdV or NoV were higher than between the two human viruses, indicating that bacteriophages are feasible indicators of human viral pathogens of fecal origin and constitute a promising, easy to use and affordable alternative to human viruses for routine water safety monitoring.

## Introduction

To date, over 150 human enteric viruses have been detected in water bodies (Rodríguez-Lázaro et al., 2012; Tran et al., 2015; Farkas et al., 2019), including adenoviruses, noroviruses, enteroviruses, sapoviruses, rotaviruses, and polyomaviruses, among others. As the assessment of all pathogenic viral strains in water is not feasible, indicators have been traditionally used as a proxy. The most used fecal indicators worldwide are Escherichia *coli* and enterococci, but they are less resistant to environmental stresses than viruses such as temperature, pH and sun irradiation and behave differently in the environment (Gerba et al., 1979; Wyer et al., 1995; Baggi et al., 2001; Borchardt et al., 2004; Harwood et al., 2005; Costán-Longares et al., 2008). As an alternative, the monitoring of reference viruses such as NoV and human adenovirus (HAdV) has been proposed (Hundesa et al., 2006; Ahmed et al., 2010; Rusiñol et al., 2014; Mayer et al., 2016). Enteroviruses are the most easily detectable enteric viruses by cell culture, and have been considered as potential indicators of the human enteric viruses (Grabow, 2007). Enteroviruses are recommended in the few water quality regulations that include virus-based criteria (EEC, 1976; USEPA, 1992). However, cell culture is expensive, time-consuming and needs specialized staff and equipment. Since the advent of genomic techniques, genome fragments of enteric viruses excreted by humans and animals have also been proposed as potential viral indicators (Hot et al., 2003; Albinana-Gimenez et al., 2009; Hunt et al., 2010; Silva et al., 2011; Wong et al., 2012). Additionally, viruses that exclusively infect humans, such as HAdV, have been postulated as microbial source tracking (MST) markers (Hundesa et al., 2006; Ahmed et al., 2010; Rusiñol et al., 2014).

However, human infectious viruses and human viral genomes both present serious drawbacks as indicators of waterborne enteric viruses. Firstly, the concentration of human viruses is variable in sewage and low in other types of water, requiring cumbersome, time-consuming and costly, concentration procedures (Hewitt et al., 2013; Haramoto et al., 2015). Secondly, there is no evidence that the presence and concentration of a given human virus in a water matrix unequivocally predicts the presence of other viruses (Irving and Smith, 1981; Chapron et al., 2000; Jiang et al., 2001). Finally, genomic methods have the inconvenience of not being able to distinguish between infectious and non-infectious viruses without additional unwieldy steps (Nuanualsuwan and Cliver, 2002; Jofre and Blanch, 2010). Such a distinction is essential when evaluating water treatments and natural inactivation and risk to human health.

Bacteriophages infecting bacteria have been proposed as efficient indicators of human viruses transmitted in water (IAWPRC, 1991; Grabow, 2004), and concurrence between enteroviruses and human-associated bacteriophages infecting *Bacteroides fragilis* has been demonstrated in a range of matrices including bivalve molluscs, treated wastewater, etc. (Tartera et al., 1988; Ebdon et al., 2007; Costán-Longares et al., 2008). Bacteriophages have been incorporated in recently developed guidelines for water quality monitoring (NHMRC, 2011; European Commission, 2018; Health Canada, 2019). In particular, somatic bacteriophages and F-RNA phages have proved to be reliable indicators of fecal viral pollution and suitable for use in water treatment processes (Jofre et al., 2016; Jebri et al., 2017). MST studies have used genogroups of F-specific RNA phages (Jofre et al., 2011) and bacteriophages infecting selected strains of *Bacteroides* (Tartera et al., 1989; Gómez-Dóñate et al., 2011; Jofre et al., 2014) to detect the source of fecal pollution in water environments. Recent studies have identified crAssphage (crAssPH) as the most abundant bacteriophage family in sewage, with *Bacteroides* as the putative host (Dutilh et al., 2014; Shkoporov et al., 2018), and its potential as a human-associated MST molecular marker is being evaluated (García-Aljaro et al., 2017; Ahmed et al., 2018; Ballesté et al., 2019). A study comparing the efficacy of human viruses with that of the molecular marker crAssPH and phages infecting the human-associated *Bacteroides* GA17 strain (GA17PH) to predict the origin of fecal pollution is therefore timely and of interest.

In this work, we determined the presence and abundance of genome fragments of HAdV, noroviruses GI and GII (NoV), crAssPH, and cultured phages infecting *Bacteroides thetaiotaomicron* strain GA17 (GA17PH), *B. fragilis* strain PG76 for porcine pollution (PGPH), and the sum of these two host-associated *Bacteroides* phages (BACPH) in human and animal wastewaters and fecal slurries in five European countries. The aim was to evaluate their sensitivity and specificity as MST markers for human pollution source detection and to assess the potential of the bacteriophages as indicators of the two human viruses (HAdV and NoV).

## Materials and Methods

### Samples and Sampling Campaigns

A total of 120 sewage and wastewater samples and animal fecal slurries were collected from municipal wastewater treatment plants, abattoirs and farms in five different countries (Austria, Finland, Germany, Portugal, and Spain) from 2013 to 2014 within the framework of the European Project AQUAVALENS. These samples were of different origins: human (35), porcine (24), and other animals (61) [bovine (23), poultry (24), horse (7), dog (2), cat (2), goat (1), bird (1), and rabbit (1)]. The sewage samples came from communities with 2,100 to 4.0 million inhabitants. Wastewater was taken from abattoirs processing about 400 pigs, 8,000 ruminant animals and 100,000 poultry per day. Animal fecal slurries were obtained by mixing feces from at least 10 individual animals with sterile water. Details of each sample can be found in Supplementary Material. All the countries took samples of the different origins throughout the year to avoid any seasonality effect. They were collected in sterile containers and kept at 4°C while in transit to the laboratory. One hundred ml of each sample was sent to the other partner institutions at 4°C to perform the assigned parameter analysis (NoV, Finland; HAdV, Portugal; PGPH, crAssPH and GA17PH, Spain) and immediately processed.

### Detection and Enumeration of MST Markers

#### Detection of Host-Associated *Bacteroides* Phages

Phages infecting human- and porcine-associated *Bacteroides* species were enumerated according to the ISO standard method 10705-4 (ISO, 2001). Plaque-forming units (PFU) of host-associated *Bacteroides* phages were enumerated by the double-agar-layer technique using the *B. thetaiotaomicron* strain GA17 to detect human pollution and *B. fragilis* strain PG76 for porcine pollution (Payan et al., 2005; Gómez-Dóñate et al., 2011). One ml of fresh samples was directly analyzed in triplicate with the corresponding host strain.

#### Detection of Human Viruses: Adenovirus and Norovirus

To detect HAdV, 200 μl of sewage samples were extracted directly using a QIAamp DNA Blood Mini Kit (Qiagen). HAdV sequences were amplified following a previously described protocol (Hernroth et al., 2002). Each run included the original sample, 10- and 100-fold dilutions of each sample, a standard curve and positive and negative controls.

Norovirus GI and GII (NoV) sequences were amplified following ISO/TS 15216-1 (ISO, 2013) with some modifications. Briefly, a sample volume of 250 μl was used for RNA extraction, which was performed using a NucliSens^®^ Magnetic Extraction Kit and NucliSens^®^ MiniMag^®^ instrument (Biomerieux, Boxtel, Netherlands) according to the manufacturer’s instructions. The sample was spiked with mengovirus to be used as a positive process control. Samples were amplified in a 20 μl-reaction using the QuantiTect Probe RT-PCR Kit (Qiagen, Hilden, Germany) and Rotorgene PCR cycler (Corbett) as described by Oristo et al. (2018), except using primer-probe sets as mentioned in ISO 15216: QNIF2 (FW) ATGTTCAGRTGGATGAGRTTCTCWGA, COG2R (REV) TCGACGCCATCTTCATTCACA, and QNIFs (PROBE) AGCACGTGGGAGGGCGATCG. Reverse transcrip tion- PCR runs included sample RNAs undiluted and diluted 1:10. For every set of samples, a negative extraction control, positive external RNA controls, and dilutions of purified plasmid dsDNA for construction of a standard curve were added.

#### Detection of Human-Associated crAssphage

DNA was extracted directly from 1 ml using the QIAamp DNA Blood Mini Kit (Qiagen). The abundance of crAssPH was analyzed with TaqMan Environmental Master Mix 2.0 (Applied Biosystems) using ABI StepOne Real-Time qPCR as previously described (García-Aljaro et al., 2017). DNA extraction controls were run together with the samples.

### Statistical Analyses

Specificity and sensitivity of the different markers in detecting fecal pollution of known human or non-human origin were calculated as follows: specificity was defined as the proportion of negative samples in which the marker was not detected (true negatives/[true negatives + false positives]), whereas sensitivity was defined as the proportion of positive samples in which the marker was detected (true positives/[true positives + false negatives]). Spearman’s correlation coefficients were used to study the relationship between the different markers. Traditional multidimensional scaling was carried out in R using the library stats (R Core Team, 2019). Receiver operating characteristic (ROC) curve analysis was performed using OptimalCutpoints R library (López-Ratón et al., 2014). ROC curve analysis was performed to define the cut-off levels for the predictors of NoV and HAdV. The criteria for obtaining the optimal cut-off point was based on the Youden index. The optimal cut-off point was defined as the point on the ROC curve nearest to the point where both the sensitivity and specificity were 1. Positive predictive values (PPV) for NoV and HAdV were calculated as the proportion of true positive results out of the number of samples with a positive result (true positives/[true positives + false positives]). Negative predictive values (NPV) were calculated as the proportion of true negative results out of the number of samples with a negative result (true negatives/[true negatives + false negatives]). The best cut-off points defined the thresholds, and thresholds were included in PPV and NPV calculations.

All the results were log transformed and the mean used for the calculations.

## Results

### Bacteriophages as Human MST Trackers

**FIGURE 1:**
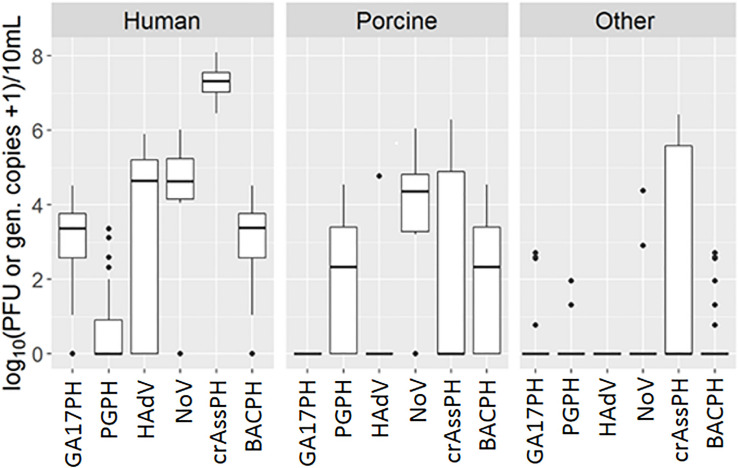
Boxplot representation of GA17PH, phages infecting human-associated *Bacteroides thetaiotaomicron* strain GA17; PGPH, phages infecting porcine-associated *B. fragilis* strain PG76; HAdV, human Adenoviruses; NoV, GI + GII Noroviruses, crAssPH, crAssphage, BACPH, sum of GA17PH, and PGPH. Outliers are shown as black dots.

Most samples of human origin tested positive for NoV, as did a similar proportion of porcine samples. Briefly, a total of 30 out of 35 human samples (85.7%) tested positive for norovirus by RT-PCR, compared to19 out of 24 porcine samples (79.2%) and only 2 out of 61 samples (3.3%) of other origins, including poultry and cow. The concentrations found in positive samples of different origin ranged from 4.05 to 6.01 log_10_ GC/10 ml (human), 3.20 to 6.04 log_10_ GC/10 ml (porcine), and 2.90 to 4.37 log_10_ GC/10 ml (other) (Figure 1 and Supplementary Table 1). Important geographical differences were observed for the specificity and sensitivity for human source detection. Whereas the NoV marker presented a sensitivity ≥80% for all the countries except Portugal, its specificity was low (71, 63, and 55% in Germany, Portugal, and Spain, respectively), mainly due to its detection in pig samples (Table 1); in contrast, specificity in Austria was 100%.

**TABLE 1:** Specificity (sp) and sensitivity (se) of the different markers at the different geographical locations in detecting human pollution (GA17PH, crAssPH, HAdV, and NoV) and porcine pollution (PGPH).

	GA17PH		PGPH		crAssPH		HAdV		NoV	
	sp	se	sp	se	sp	se	sp	se	sp	se
Austria	1.00	0.88	0.79	0.00	1.00	1.00	1.00	0.88	1.00	1.00
Germany	0.93	1.00	0.93	0.00	1.00	1.00	0.93	0.60	0.71	0.80
Finland	1.00	0.86	0.94	1.00	1.00	1.00	1.00	0.71	0.82	1.00
Portugal	0.88	1.00	0.94	1.00	0.38	1.00	1.00	0.14	0.63	0.57
Spain	0.95	0.83	0.65	1.00	0.10	1.00	1.00	0.67	0.55	1.00

*GA17PH, phages infecting human-associated Bacteroides thetaiotaomicron strain GA17; PGPH, phages infecting porcine-associated B. fragilis strain PG76; BACPH, sum of GA17PH and PGPH; crAssPH, crAssphage; HAdV, human Adenoviruses; and NoV, GI + GII Noroviruses.*

Human adenovirus was detected almost exclusively in samples of human origin, with the exception of 1 positive porcine sample out of 24. Nevertheless, only 20 out of 33 (60.6%) human samples were positive by qPCR (the remaining 2 samples up to 35 were not analyzed due to technical problems). The concentrations in the positive samples were high, ranging from 4.29 to 5.90 log_10_ GC/10 ml (Figure 1 and Supplementary Table 1). Once again, geographical differences were observed. In this case, although the specificity of the HAdV marker for human source detection was very high in all the countries (≥90%), its sensitivity was low, being ≤80% in all the countries except Austria (Table 1).

The performance of two human-related bacteriophages (GA17PH and crAssPH) was also assessed in terms of specificity and sensitivity for human source detection. The GA17PH marker displayed a higher specificity and sensitivity for human sources than either NoV or HAdV. Accordingly, GA17PH was detected in most of the human samples (32 out of 35, 91.4%), not detected in any porcine sample, and rarely detected in the samples of mixed animal origin (4 out of 61). The concentration in the positive human source samples ranged from 1.00 to 4.52 log_10_ PFU/10 ml, whereas in the other animal wastewater samples it was 2 log_10_ units lower in those with a higher fecal load (Figure 1 and Supplementary Table 1). No geographical differences were observed for GA17PH, with specificity ≥88% and sensitivity ≥83% for human source detection (Table 1). On the other hand, crAssPH was present at higher levels in human samples, which ranged from 6.45 to 8.10 log_10_ GC/10 ml, although it was also detected in 11 out of 24 samples of porcine origin and in 17 out of 61 samples of other animals (Figure 1 and Supplementary Table 1). This marker exhibited a notable geographical variability in specificity, which was 100% for the Northern European countries, yet very low in Portugal and Spain (38 and 10%, respectively; Table 1).

As stated above, most false positives in the NoV assay were due to interference from NoV of porcine origin. These viruses may also infect humans and therefore, it could be advantageous to have a suite of markers able to predict the presence of NoV of both origins. In this study, the sum of the human-associated (GA17PH) and porcine-associated (PGPH) *Bacteroides* phages was assessed as an indicator for the presence of NoV of either human or porcine origin. It should be noted that the results of the PGPH marker showed considerable geographical variability (Table 1), with a sensitivity of 0% in Austria and Germany, where it was not detected, and a specificity of 65% in Spain. The concentration of PGPH in positive samples of wastewater and slurries ranged from 0.70 to 4.55 log_10_ PFU/10 ml.

### Bacteriophages as Indicators of HAdV and NoV

**FIGURE 2:**
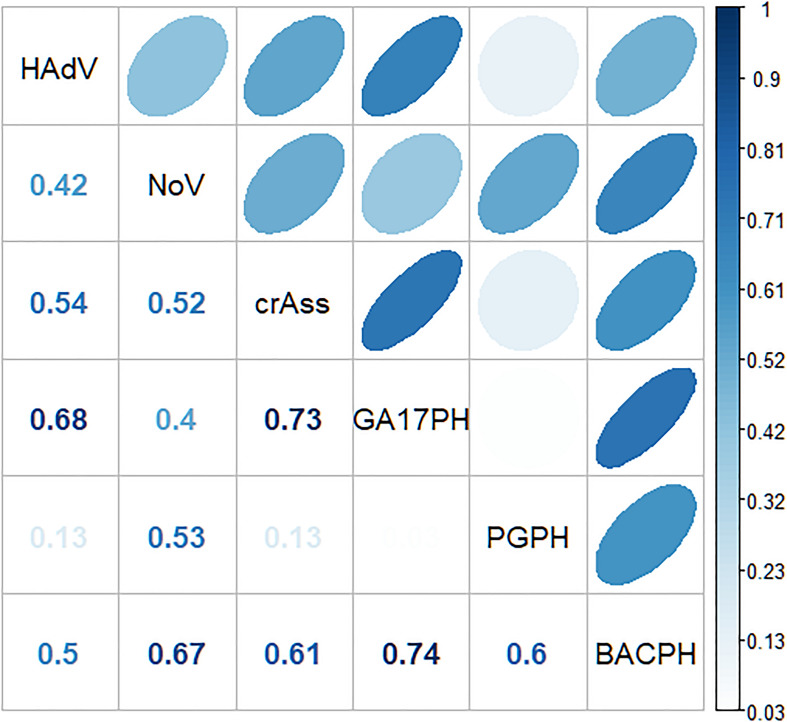
Spearman’s correlation coefficients between the different markers (GA17PH, phages infecting human-associated *Bacteroides thetaiotaomicron* strain GA17; PGPH, phages infecting porcine-associated *B. fragilis* strain PG76; BACPH, sum of GA17PH and PGPH; crAssPH, crAssphage; HAdv, human Adenoviruses; and NoV, GI + GII Noroviruses).

A significant correlation was observed between GA17PH and HAdV (*p* < 0.01, Spearman correlation coefficient rho = 0.66), as well as between crAssPH and both human viruses (*p* < 0.01, rho = 0.55, and 0.54 for HAdV and NoV, respectively; Figure 2). The sum of the two *Bacteroides* markers (GA17PH + PGPH, indicated as BACPH) gave a higher correlation with NoV (rho = 0.7) but lower with HAdV (rho = 0.44). NoV correlated more strongly with BACPH than with HAdV (rho = 0.44), indicating a weak correlation between the viruses.

**FIGURE 3:**
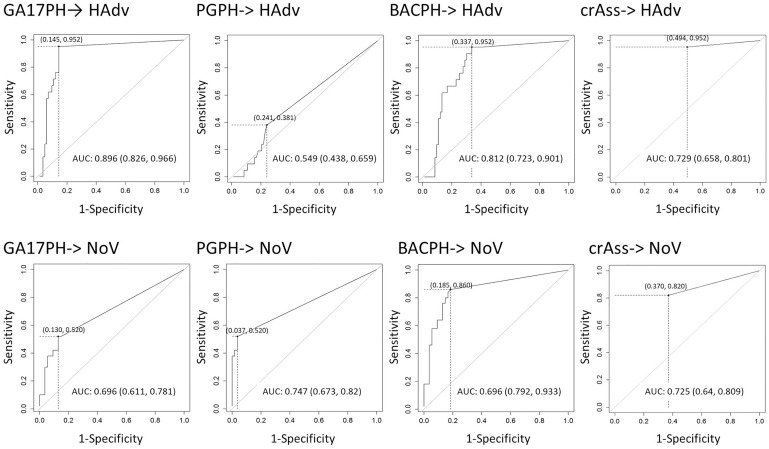
ROC curve analysis with the prediction of HAdV and NoV by the different markers (GA17PH, phages infecting human-associated *Bacteroides thetaiotaomicron* strain GA17; PGPH, phages infecting porcine-associated *B. fragilis* strain PG76; BACPH, sum of GA17PH and PGPH; crAssPH, crAssphage; HAdv, human Adenoviruses; and NoV, GI + GII Noroviruses). AUC with the log of the minimal concentration (in brackets) needed to predict HAdV and NoV are indicated in the table below.

In view of these results, the concentration limits of the different bacteriophage markers that would be required to predict the presence of the enteric viruses were assessed statistically with the analysis of ROC curves (Figure 3). Based on the ROC curve analyses, the minimum concentration of crAssPH and GA17PH needed to predict HAdV was 1.00 and 1.54 log_10_ units, respectively, which was slightly higher than the respective 1.00 and 1.04 log_10_ units required to predict NoV. GA17PH showed a PPV of 63% and NPV of 99% for HAdV; for NoV a higher PPV of 79% and a lower NPV of 66% were obtained (Table 2). The sum of the markers PGPH and GA17PH (BACPH) increased the PPV and NPV for NoV to 81 and 86%, respectively, but gave a lower PPV of 42% for HAdV. In the case of crAssPH, a higher NPV of 79% was obtained for NoV, but the PPV was lower for both HAdV and NoV, being 33 and 67%, respectively. In summary, the highest PPV and NPV for HAdV were obtained with GA17PH (63 and 99%, respectively) followed by crAssPH (33 and98%, respectively), whereas for NoV the most acceptable PPV and NPV were obtained with BACPH (81 and 86%, respectively).

**TABLE 2:** Predictive capacity of the different markers for HAdV and NoV.

Markers	GA17PH		PGPH		BACPH		crAssPH	
	PPV	NPV	PPV	NPV	PPV	NPV	PPV	NPV
HAdV	0.63	0.99	0.29	0.83	0.42	0.98	0.33	0.98
NoV	0.79	0.66	0.93	0.68	0.81	0.86	0.67	0.79

*Positive predictive value (PPV), negative predictive value (NPV). GA17PH, phages infecting human-associated Bacteroides thetaiotaomicron strain GA17; PGPH, phages infecting porcine-associated B. fragilis strain PG76; BACPH, sum of GA17PH and PGPH; crAssPH, crAssphage; HAdV, human Adenoviruses; and NoV, GI + GII Noroviruses.*

## Discussion

In the current work, we assessed the suitability of human viruses (NoV and HadV) as markers of fecal viral pollution (Albinana-Gimenez et al., 2009; Rusiñol et al., 2014), and compared their performance with that of different source-associated *Bacteroides* phages (Gómez-Dóñate et al., 2011), including the recently reported human bacteriophage crAssPH (García-Aljaro et al., 2017; Stachler et al., 2017; Ballesté et al., 2019).

In this study, HAdV was detected in 60% of the human sewage samples, NoV in 80%, GA17PH in 91%, and crAssPH in 100%, indicating that the bacteriophage markers were more sensitive than the enteric viruses. Comparable with our results, studies in Japan and New Zealand did not detect NoV in 10–40% of raw sewage samples, and also report a variable detection (Wolf et al., 2010; Hewitt et al., 2013; Haramoto et al., 2015). On the other hand, high sensitivity and specificity for human source detection has been generally observed for HAdV (Albinana-Gimenez et al., 2009; Ahmed et al., 2010; Rodríguez-Lázaro et al., 2012; Hewitt et al., 2013; Rusiñol et al., 2014; Haramoto et al., 2015), leading to its proposal as a human MST marker. Here, although HAdV specificity was high, sensitivity was only 60%, with variability among geographical areas. Another study has also reported lower specificity in individual human feces or septic tanks and sewage, which could be attributed to the variable prevalence of HAdV in the population pool, and the variety of HAdV species associated with outbreaks (Wolf et al., 2010). Another explanation for the differences among studies could be the different volumes and concentrating methods used. In our study, HAdV was measured directly from a low volume (0.2 ml) of sewage, but as no concentration method was required, DNA loss due to concentration was avoided. It has to be pointed out that in our study GA17PH and PGPH were detected by culture-based methods, whereas NoV and AdV were detected by qPCR. In theory, qPCR analysis of the two *Bacteroides* phages would provide useful data, however, it was not possible to perform because there are not available qPCR methods for these phages since they are a very heterogeneous group and difficult to analyze them by this technique.

The arithmetic mean of NoV GI and GII combined in samples was 4.79 log_10_ GC/10 ml. Values reported in the literature are highly variable, ranging from 1 to 7 log_10_ GC/10 ml (Kitajima et al., 2012; Hewitt et al., 2013; Haramoto et al., 2015; Katayama and Vinjé, 2017). Such variability hampers NoV detection in the environment and its application as an MST marker, another disadvantage being its presence in pig feces in some areas in this study. The mean of HAdV in raw sewage (5.13 log_10_ GC/10 ml) was found to be higher than in other studies (about 3–4 log_10_ GC/1,000 ml; Hewitt et al., 2013; Rusiñol et al., 2014; Haramoto et al., 2015), although a considerable variability (2–7 log_10_ GC/10 ml) between geographical areas has also been observed (Allard and Vantarakis, 2017). As a marker, HAdV showed good specificity for human source detection, being found mainly in human fecal samples, but its low sensitivity limits its use for MST unless combined with more sensitive markers (Blanch et al., 2006). To overcome the limitations of viruses as MST markers, host-associated bacteriophages have been proposed as MST tools, such as human-associated *Bacteroides* strains GA17 and GB124, the animal-associated PG76 (Ebdon et al., 2007; Gómez-Dóñate et al., 2011), or crAssPH (García-Aljaro et al., 2017), recently related to human pollution. In this study, we assessed the capacity of human phages infecting the human *B. thetaiotaomicron* GA17 strain as well as the porcine strain PG76 to predict the presence of human viruses. The GA17PH marker displayed the highest sensitivity and specificity, giving values from 1 to 4.52 log_10_ PFU/10 ml, in accordance with previous reports of 1.69 to 5.84 PFU/100 ml in Spain, Sweden, France, United Kingdom, Tunisia, and Colombia (Payan et al., 2005; Blanch et al., 2006; Venegas et al., 2015; Yahya et al., 2015). CrAssPH was sensitive, although its specificity varied according to location. The concentration of crAssPH found in our study was around 10^7^ GC/10 ml, which was 2.5 log_10_ units higher compared to NoV. These results are in accordance with those of Farkas and co-workers, who compared crAssPH with several enteric viruses (NoVGI, NoVGII, SaV, AdV, and JCV; Farkas et al., 2019). However, their crAssPH numbers only correlated with JCV and not with any of the human viruses analyzed this study. The low concentration of GA17PH in raw sewage may be a problem for the analysis of environmental samples where fecal pollution is diluted. However, as the concentration of crAssPH in river water is 2–3 log10 units higher than GA17PH, the two markers could be used in combination in certain geographical locations or in catchments not containing large numbers of pigs or abattoirs (Ballesté et al., 2019).

It should be noted that most of the false positives of NoV as an MST marker of human fecal contamination were due to the cross-reaction of the assay with NoV of porcine origin. A possible explanation is that NoV GII real-time RT-PCR primers and probes could detect porcine GII NoV strains, since primers and probes used in the RT-qPCR assay are highly similar to the sequences available in GeneBank for porcine NoV (LC509111, AB126329, HQ392821, and AY823305). In fact, strong homologies between swine NoVs genomic sequences and human primer sequences have been reported previously (L’Homme et al., 2009). Although human infections with porcine GII NoV have not been documented (Villabruna et al., 2019), they cannot be excluded. On the other hand, pigs may be susceptible to infection with human NoV strains, which have been detected in porcine feces (Mattison et al., 2007; Sisay et al., 2016), although how commonly this occurs is not known. Given the risk of these viruses infecting humans, it would be of interest to have a suite of markers able to predict the presence of NoV of both origins (Mattison et al., 2007). In this case, the combination of GA17PH and PGPH (BACPH) could be used as an indicator for the presence of NoV of either human or porcine origin. Nevertheless, it should be noted that the PGPH marker showed geographical variability, as reported for other *Bacteroides* phages, and thus a *Bacteroides* pig-associated strain may be isolated in each targeted area (Payan et al., 2005) or other MST marker capable of identifying porcine sources such as Bacteroidales PG markers.

In summary, the results presented here show that bacteriophages infecting human-associated *Bacteroides* strains are a promising alternative for the prediction of human viruses of fecal origin as they outperform human viruses such as AdV and NoV, and provide an easier and more affordable technique for routine monitoring, avoiding the need to look for pathogenic viruses. Moreover, they also provide useful information on infectiousness/viability of the phages which is useful when establishing risk to human health.

## Data Availability Statement

The raw data supporting the conclusions of this article will be made available by the authors, without undue reservation.

## Author Contributions

EB, LS-C, CG-A, SM, and LM participated in the sampling and carried out the experiments. JM performed the statistical analyses. SM, LM, AB, AF, and AT conceived and supervised the sampling experiments in the different laboratories. EB, JJ, and CG-A contributed to the data analysis and writing of the manuscript. All authors revised the manuscript.

## Conflict of Interest

The authors declare that the research was conducted in the absence of any commercial or financial relationships that could be construed as a potential conflict of interest. The reviewer, JL, declared a past collaboration with one of the authors, LM.
