# Supplementary material for: Bacteriophages Are Good Estimators of Human Viruses Present in Water
Source: Front Microbiol. 2021 May 3;12:619495. doi: 10.3389/fmicb.2021.619495 (PMC8128106; doi:10.3389/fmicb.2021.619495)
Supplement: Supplementary file 1 [file Table_1.DOCX]

**Supplementary Table 1** Descriptive statistics of GA17PH, phages infecting human specific *Bacteroides thetaiotaomicron* strain GA17: A) Human; B) Porcine; C) Other animal origina; PGPH, phages infecting porcin specific *B. fragilis* strain PG76; BACPH, sum of GA17PH and PGPH; crAssPH, crAssphage; HAdV, human Adenoviruses; NoV, GI+GII Noroviruses. Na, data not available

1. Human

|  | GA17PH | PGPH | BACPH | crAssPH | AdV | | NoV | |
| --- | --- | --- | --- | --- | --- | --- | --- | --- |
| Num of positive samples / Total samples | 32/35 | 10/35 | 33/35 | 33/33 | 20/33 | 30/35 | |  |
| mean | 3.24 | 1.95 | 3.22 | 7.30 | 5.13 | 4.79 | |  |
| Sd | 0.79 | 0.91 | 0.82 | 0.41 | 0.46 | 0.54 | |  |
| Min | 1.00 | 0.70 | 1.00 | 6.45 | 4.29 | 4.05 | |  |
| Max | 4.52 | 3.36 | 4.52 | 8.10 | 5.90 | 6.01 | |  |

1. Porcine

|  | GA17PH | PGPH | BACPH | crAssPH | HAdV | NoV |
| --- | --- | --- | --- | --- | --- | --- |
| Num of positive samples / Total samples | 0/24 | 16/24 | 16/24 | 11/24 | 1/24 | 19/24 |
| mean | Na | 3.05 | 3.05 | 5.03 | 4.77 | 4.58 |
| Sd | Na | 1.17 | 1.17 | 0.59 | Na | 0.78 |
| Min | Na | 0.70 | 0.70 | 4.26 | 4.77 | 3.20 |
| Max | Na | 4.55 | 4.55 | 6.29 | 4.77 | 6.04 |

1. Other animal origin

|  | GA17PH | PGPH | BACPH | crAssPH | HAdV | NoV |
| --- | --- | --- | --- | --- | --- | --- |
| Num of positive samples / Total samples | 4/61 | 2/61 | 6/61 | 17/61 | 0/61 | 2/61 |
| mean | 2.14 | 1.63 | 1.97 | 5.63 | Na | 3.64 |
| Sd | 0.96 | 0.46 | 0.82 | 0.62 | Na | 1.04 |
| min | 0.70 | 1.30 | 0.70 | 4.21 | Na | 2.90 |
| max | 2.72 | 1.95 | 2.72 | 6.42 | Na | 4.37 |

**Supplementary Table 2.** Spearman correlation coefficients. GA17PH, phages infecting human specific *Bacteroides thetaiotaomicron* strain GA17; PGPH, phages infecting porcin specific *B. fragilis* strain PG76; BACPH, sum of GA17PH and PGPH; crAssPH, crAssphage; HAdv, human Adenoviruses; NoV, GI+GII Noroviruses.

|  | GA17PH | PGPH | BACPH | crAssPH | AdV | NoV |
| --- | --- | --- | --- | --- | --- | --- |
| GA17PH | - | >0.05 | <0.01 | <0.01 | <0.01 | <0.01 |
| PGPH | 0.034 | - | <0.01 | 0.02 | >0.05 | <0.01 |
| BACPH | 0.739 | 0.605 | - | <0.01 | <0.01 | <0.01 |
| crAssPH | 0.703 | 0.221 | 0.648 | - | 0.551 | 0.542 |
| AdV | 0.663 | 0.133 | 0.488 | <0.01 | - | <0.01 |
| NoV | 0.443 | 0.559 | 0.704 | <0.01 | 0.439 | - |

**Supplementary Table 3.** Roc curves analysis showing AUC parameter and the log of the minimal concentration of each parameter needed for the prediction of HAdv or NoV (in brackets).

| Markers | GA17PH | PGPH | BACPH | crAssPH |
| --- | --- | --- | --- | --- |
| HAdV | 0.896 (1.54) | 0.549 (0.78) | 0.812 (1.54) | 0.729 (1.00) |
| NoV | 0.696 (1.04) | 0.747 (0.78) | 0.863 (0.78) | 0.725 (1.00) |
